# 2-Methyl-4,4-dioxo-*N*-phenyl-5,6-di­hydro-1,4-oxathiine-3-carboxamide (Oxycarboxin)

**DOI:** 10.1107/S1600536810037669

**Published:** 2010-09-30

**Authors:** J. Emery Brown, Russell G. Baughman

**Affiliations:** a Department of Chemistry, Truman State University, Kirksville, MO 63501-4221, USA

## Abstract

In the title compound, C_12_H_13_NO_4_S, a systemic fungicide, the heterocycle adopts a lounge chair conformation and the dihedral angle between the ring planes is 25.8 (2)°. Inter­molecular C—H⋯O hydrogen bonds are noted in the crystal structure. Also observed is a short inter­action of a methyl­ene hydrogen atom with the π-electron system of a phenyl ring in an adjacent mol­ecule.

## Related literature

The title structure was determined as part of a larger project involving the structures of fungicides, see: Baughman & Paulos (2005 ▶). For the mode of biological action of the title compound, see: Ulrich & Mathre (1972 ▶).
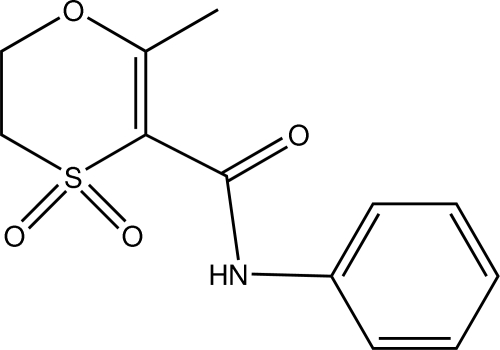


## Experimental

### 

#### Crystal data

C_12_H_13_NO_4_S
*M* *_r_* = 267.29
Triclinic, 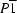

*a* = 5.9985 (4) Å
*b* = 8.3178 (6) Å
*c* = 13.1333 (8) Å
α = 104.702 (4)°
β = 93.180 (5)°
γ = 106.876 (5)°
*V* = 600.59 (7) Å^3^
*Z* = 2
Mo *K*α radiation
μ = 0.28 mm^−1^
*T* = 295 K
0.54 × 0.44 × 0.16 mm

#### Data collection

Bruker P4 diffractometer
Absorption correction: integration (*XSHELL*; Bruker, 1999 ▶) *T*_min_ = 0.888, *T*_max_ = 0.959
2152 measured reflections
2152 independent reflections
1884 reflections with *I* > 2σ(*I*)
3 standard reflections every 100 reflections  intensity decay: 1.2%

#### Refinement

*R*[*F* ^2^ > 2σ(*F* ^2^)] = 0.049
*wR*(*F* ^2^) = 0.148
*S* = 1.17
2152 reflections
165 parameters
H-atom parameters constrained
Δρ_max_ = 0.26 e Å^−3^
Δρ_min_ = −0.30 e Å^−3^

### 

Data collection: *XSCANS* (Bruker, 1996 ▶); cell refinement: *XSCANS*; data reduction: *XSCANS*; program(s) used to solve structure: *SHELXS86* (Sheldrick, 2008 ▶); program(s) used to refine structure: *SHELXL97* (Sheldrick, 2008 ▶); molecular graphics: *SHELXTL/PC* (Sheldrick, 2008 ▶); software used to prepare material for publication: *SHELXTL/PC*, *SHELXL97* and *PLATON* (Spek, 2009 ▶).

## Supplementary Material

Crystal structure: contains datablocks I, global. DOI: 10.1107/S1600536810037669/fb2200sup1.cif

Structure factors: contains datablocks I. DOI: 10.1107/S1600536810037669/fb2200Isup2.hkl

Additional supplementary materials:  crystallographic information; 3D view; checkCIF report

## Figures and Tables

**Table 1:** Hydrogen-bond geometry (Å, °) *Cg* is the centroid of the C7–C12 ring.

*D*—H⋯*A*	*D*—H	H⋯*A*	*D*⋯*A*	*D*—H⋯*A*
N1—H1*A*⋯O3	0.86	2.09	2.819 (4)	142
C2—H2*B*⋯O2^i^	0.97	2.50	3.274 (5)	137
C5—H5*A*⋯O4^ii^	0.96	2.52	3.422 (6)	157
C9—H9⋯O4^iii^	0.93	2.49	3.419 (5)	175
C1—H1*C*⋯*Cg*^iv^	0.97	2.82	3.645	144

Symmetry codes: (i) 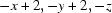; (ii) 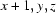; (iii) 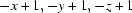; (iv) 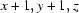.

**Fig. 1.**
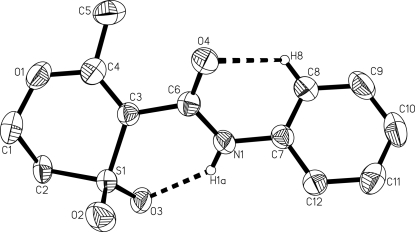
The title molecule showing the labeling of the non-H atoms. The displacement ellipsoids are drawn at the 50% probability levels; the H atoms are drawn as small spheres of arbitrary radius. Intramolecular hydrogen bonds involving H1a and H8 are indicated by dashed lines.

## supplementary crystallographic information

### Comment

The crystal structure of 2,3-dihydro-5-carboxanilide-6-methyl-1,4-oxathiin-4,4-dioxide (oxycarboxin, also known as Plantvax^R^) was determined as part of a larger project involving the structures of fungicides (Baughman & Paulos, 2005). It is the dioxide form of its parent compound, carboxin, and is a member of the oxathiin class of systemic (works from within the plant system) fungicides. The mode of action of this class of compounds is to inhibit succinate oxidation of succinate dehydrogenase in the fungal class *Basiomycetes* (Ulrich & Mathre, 1972).

The molecules of the title structure (Fig. 1) are interconnected by intermolecular hydrogen bonds, while the molecule itself contains intramolecular hydrogen bonds (Tab. 1). The N1—H1A···O3—S1 and the C8—H8···O4—C6 intramolecular hydrogen bonds restrict all of the torsion angles around the N1—C6, N1—C7, and C3—C6 bonds. H1C is observed to be interacting with the π-electron system of the adjacent phenyl ring at 1 + *x*, 1 + *y*, *z* (Fig. 1, Tab. 1).

The C1//C2//S1//C3//C4//O1 ring is nonplanar; C1 and C2 are located +0.435 (6) and -0.398 (6) Å, respectively, from the 4-membered planar S1//C3//C4//O4 group, which has an r.m.s. deviation from the mean plane equal to 0.030 Å.

### Experimental

A 99.9% pure sample of the title compound was purchased from Sigma-Aldrich. Crystals were grown by slow evaporation of a solution in EtOH.

### Refinement

Approximate positions for all H atoms were first obtained from a difference electron density map. However, the hydrogens were situated into idealized positions and the H-atoms have been refined within the riding atom approximation. The constraints used: C_aryl_-H_aryl_=0.93; C_methyl_-H_methyl_=0.96; C_methylene_-H_methylene_=0.97 and N_sec. amine_—H_sec. amine_=0.86 Å. The idealized methyl group was allowed to rotate about the C—C bond during the refinement [AFIX 137; *SHELXL97* (Sheldrick, 2008)]. *U*_iso_(H_methyl_)=1.5*U*_eq_(C_methyl_) or *U*_iso_(H_aryl/methylene_/N)=1.2*U*_eq_(C_aryl/methylene/N_).

*PLATON* (Spek, 1999) indicated the presence of a possible twin by reticular non-merohedry or accidental intergrowth and suggested the twinning matrix that corresponded to the rotation by 180° about the direction [1 2 4] quasiperpendicular to (0 0 1): (h2,k2,l2)=(h1,k1,l1)× (-1.000,0.000,0.618//0.000,-1.000,0.931//0.000,0.000,1.000). The number of the overlapped reflections equals to 366. The minor-domain fraction was refined to 0.083 (5).

### Crystal data

**Table:**

C_12_H_13_NO_4_S	*Z* = 2
*M**_r_* = 267.29	*F*(000) = 280
Triclinic, *P*1	*D*_x_ = 1.478 Mg m^−^^3^
Hall symbol: -P 1	Mo *K*α radiation, λ = 0.71073 Å
*a* = 5.9985 (4) Å	Cell parameters from 100 reflections
*b* = 8.3178 (6) Å	θ = 10.7–18.8°
*c* = 13.1333 (8) Å	µ = 0.28 mm^−^^1^
α = 104.702 (4)°	*T* = 295 K
β = 93.180 (5)°	Parrallelepiped, colorless
γ = 106.876 (5)°	0.54 × 0.44 × 0.16 mm
*V* = 600.59 (7) Å^3^	

### Data collection

**Table:**

Bruker P4 diffractometer	1884 reflections with *I* > 2σ(*I*)
Radiation source: normal-focus sealed tube	*R*_int_ = 0.0000
graphite	θ_max_ = 25.3°, θ_min_ = 2.7°
θ/2θ scans	*h* = −7→7
Absorption correction: integration (*XSHELL*; Bruker, 1999)	*k* = −9→9
*T*_min_ = 0.888, *T*_max_ = 0.959	*l* = −7→15
2152 measured reflections	3 standard reflections every 100 reflections
2152 independent reflections	intensity decay: 1.2%

### Refinement

**Table:**

Refinement on *F*^2^	Primary atom site location: structure-invariant direct methods
Least-squares matrix: full	Secondary atom site location: difference Fourier map
*R*[*F*^2^ > 2σ(*F*^2^)] = 0.049	Hydrogen site location: difference Fourier map
*wR*(*F*^2^) = 0.148	H-atom parameters constrained
*S* = 1.17	*w* = 1/[σ^2^(*F*_o_^2^) + (0.0317*P*)^2^ + 1.2476*P*] where *P* = (*F*_o_^2^ + 2*F*_c_^2^)/3
2152 reflections	(Δ/σ)_max_ < 0.001
165 parameters	Δρ_max_ = 0.26 e Å^−^^3^
0 restraints	Δρ_min_ = −0.30 e Å^−^^3^
51 constraints	

### Special details

**Table:**

Geometry. All s.u.'s (except the s.u. in the dihedral angle between two l.s. planes) are estimated using the full covariance matrix. The cell s.u.'s are taken into account individually in the estimation of s.u.'s in distances, angles and torsion angles; correlations between s.u.'s in cell parameters are only used when they are defined by crystal symmetry. An approximate (isotropic) treatment of cell s.u.'s is used for estimating s.u.'s involving l.s. planes.
Refinement. Refinement of *F*^2^ against ALL reflections. The weighted *R*-factor, *wR*, and goodness of fit, *S*, are based on *F*^2^, conventional *R*-factors, *R*, are based on *F*, with *F* set to zero for negative *F*^2^. The threshold expression of *F*^2^ > 2σ(*F*^2^) is used only for calculating *R*-factors(gt) *etc*. and is not relevant to the choice of reflections for refinement. *R*-factors based on *F*^2^ are statistically about twice as large as those based on *F*, and *R*-factors based on ALL data will be even larger.

### Fractional atomic coordinates and isotropic or equivalent isotropic displacement parameters (Å^2^)

**Table:**

	*x*	*y*	*z*	*U*_iso_*/*U*_eq_	
S1	0.86650 (16)	0.76152 (12)	0.09237 (7)	0.0340 (3)	
O1	1.3241 (5)	0.9576 (4)	0.2477 (2)	0.0476 (7)	
O2	0.7405 (5)	0.8747 (4)	0.0705 (3)	0.0533 (8)	
O3	0.7619 (5)	0.5767 (4)	0.0397 (2)	0.0455 (7)	
O4	0.7181 (5)	0.7679 (4)	0.3786 (2)	0.0483 (7)	
N1	0.5358 (5)	0.5725 (4)	0.2219 (2)	0.0377 (7)	
H1A	0.5393	0.5414	0.1546	0.045*	
C1	1.2901 (7)	0.9966 (5)	0.1490 (4)	0.0467 (10)	
H1B	1.4418	1.0488	0.1291	0.056*	
H1C	1.2046	1.0808	0.1576	0.056*	
C2	1.1548 (7)	0.8332 (5)	0.0621 (3)	0.0379 (9)	
H2A	1.2291	0.7431	0.0584	0.045*	
H2B	1.1517	0.8584	−0.0060	0.045*	
C3	0.9171 (6)	0.7890 (5)	0.2294 (3)	0.0318 (8)	
C4	1.1355 (7)	0.8708 (5)	0.2853 (3)	0.0386 (9)	
C5	1.2078 (8)	0.8729 (7)	0.3958 (3)	0.0520 (11)	
H5A	1.3705	0.8796	0.4045	0.078*	
H5B	1.1134	0.7677	0.4095	0.078*	
H5C	1.1861	0.9727	0.4449	0.078*	
C6	0.7168 (6)	0.7096 (5)	0.2834 (3)	0.0344 (8)	
C7	0.3405 (7)	0.4774 (5)	0.2627 (3)	0.0339 (8)	
C8	0.3722 (8)	0.4250 (6)	0.3528 (3)	0.0459 (10)	
H8	0.5224	0.4541	0.3891	0.055*	
C9	0.1786 (9)	0.3287 (6)	0.3887 (4)	0.0555 (12)	
H9	0.1990	0.2953	0.4502	0.067*	
C10	−0.0441 (9)	0.2821 (6)	0.3339 (4)	0.0574 (12)	
H10	−0.1735	0.2167	0.3577	0.069*	
C11	−0.0730 (8)	0.3329 (6)	0.2440 (4)	0.0508 (11)	
H11	−0.2229	0.3012	0.2068	0.061*	
C12	0.1177 (7)	0.4309 (5)	0.2077 (3)	0.0375 (8)	
H12	0.0960	0.4652	0.1467	0.045*	

### Atomic displacement parameters (Å^2^)

**Table:**

	*U*^11^	*U*^22^	*U*^33^	*U*^12^	*U*^13^	*U*^23^
S1	0.0286 (5)	0.0390 (5)	0.0340 (5)	0.0066 (4)	0.0065 (4)	0.0136 (4)
O1	0.0300 (14)	0.0505 (17)	0.0492 (17)	0.0020 (12)	0.0030 (12)	0.0044 (14)
O2	0.0463 (17)	0.071 (2)	0.0596 (19)	0.0272 (16)	0.0125 (14)	0.0363 (17)
O3	0.0411 (15)	0.0447 (16)	0.0344 (14)	−0.0056 (12)	0.0054 (12)	0.0049 (12)
O4	0.0473 (17)	0.0546 (18)	0.0334 (15)	0.0067 (14)	0.0095 (12)	0.0059 (13)
N1	0.0374 (17)	0.0426 (18)	0.0281 (15)	0.0051 (14)	0.0082 (13)	0.0091 (13)
C1	0.038 (2)	0.036 (2)	0.059 (3)	0.0018 (17)	0.0134 (19)	0.0106 (19)
C2	0.034 (2)	0.035 (2)	0.043 (2)	0.0063 (16)	0.0153 (16)	0.0115 (16)
C3	0.0314 (18)	0.0325 (18)	0.0314 (18)	0.0101 (15)	0.0059 (15)	0.0086 (15)
C4	0.036 (2)	0.036 (2)	0.041 (2)	0.0123 (16)	0.0089 (16)	0.0028 (16)
C5	0.043 (2)	0.068 (3)	0.038 (2)	0.019 (2)	−0.0017 (18)	0.002 (2)
C6	0.0335 (19)	0.0348 (19)	0.035 (2)	0.0106 (16)	0.0060 (15)	0.0103 (16)
C7	0.038 (2)	0.0308 (18)	0.0303 (18)	0.0076 (15)	0.0073 (15)	0.0068 (15)
C8	0.045 (2)	0.052 (2)	0.037 (2)	0.0065 (19)	0.0042 (18)	0.0171 (18)
C9	0.070 (3)	0.052 (3)	0.040 (2)	0.005 (2)	0.016 (2)	0.021 (2)
C10	0.058 (3)	0.050 (3)	0.057 (3)	0.002 (2)	0.027 (2)	0.017 (2)
C11	0.039 (2)	0.044 (2)	0.059 (3)	0.0048 (19)	0.008 (2)	0.007 (2)
C12	0.040 (2)	0.0339 (19)	0.0351 (19)	0.0086 (16)	0.0053 (16)	0.0072 (16)

### Geometric parameters (Å, °)

**Table:**

S1—O2	1.438 (3)	C3—C6	1.501 (5)
S1—O3	1.445 (3)	C4—C5	1.486 (6)
S1—C3	1.754 (4)	C5—H5A	0.9600
S1—C2	1.761 (4)	C5—H5B	0.9600
O1—C4	1.346 (5)	C5—H5C	0.9600
O1—C1	1.432 (5)	C7—C12	1.382 (5)
O4—C6	1.220 (4)	C7—C8	1.383 (5)
N1—C6	1.359 (5)	C8—C9	1.388 (6)
N1—C7	1.425 (5)	C8—H8	0.9300
N1—H1A	0.8600	C9—C10	1.380 (7)
C1—C2	1.509 (5)	C9—H9	0.9300
C1—H1B	0.9700	C10—C11	1.369 (7)
C1—H1C	0.9700	C10—H10	0.9300
C2—H2A	0.9700	C11—C12	1.385 (6)
C2—H2B	0.9700	C11—H11	0.9300
C3—C4	1.357 (5)	C12—H12	0.9300
			
O2—S1—O3	116.48 (19)	C4—C5—H5A	109.5
O2—S1—C3	110.85 (18)	C4—C5—H5B	109.5
O3—S1—C3	108.26 (17)	H5A—C5—H5B	109.5
O2—S1—C2	108.98 (18)	C4—C5—H5C	109.5
O3—S1—C2	109.37 (18)	H5A—C5—H5C	109.5
C3—S1—C2	101.89 (18)	H5B—C5—H5C	109.5
C4—O1—C1	118.9 (3)	O4—C6—N1	122.0 (3)
C6—N1—C7	123.8 (3)	O4—C6—C3	120.6 (3)
C6—N1—H1A	118.1	N1—C6—C3	117.4 (3)
C7—N1—H1A	118.1	C12—C7—C8	120.1 (4)
O1—C1—C2	111.0 (3)	C12—C7—N1	118.7 (3)
O1—C1—H1B	109.4	C8—C7—N1	121.2 (4)
C2—C1—H1B	109.4	C7—C8—C9	119.6 (4)
O1—C1—H1C	109.4	C7—C8—H8	120.2
C2—C1—H1C	109.4	C9—C8—H8	120.2
H1B—C1—H1C	108.0	C10—C9—C8	120.4 (4)
C1—C2—S1	107.8 (3)	C10—C9—H9	119.8
C1—C2—H2A	110.2	C8—C9—H9	119.8
S1—C2—H2A	110.2	C11—C10—C9	119.4 (4)
C1—C2—H2B	110.2	C11—C10—H10	120.3
S1—C2—H2B	110.2	C9—C10—H10	120.3
H2A—C2—H2B	108.5	C10—C11—C12	121.0 (4)
C4—C3—C6	120.0 (3)	C10—C11—H11	119.5
C4—C3—S1	121.1 (3)	C12—C11—H11	119.5
C6—C3—S1	118.8 (3)	C7—C12—C11	119.5 (4)
O1—C4—C3	125.3 (4)	C7—C12—H12	120.3
O1—C4—C5	109.0 (3)	C11—C12—H12	120.3
C3—C4—C5	125.7 (4)		
			
C4—O1—C1—C2	−53.6 (5)	C7—N1—C6—O4	−4.3 (6)
O1—C1—C2—S1	67.6 (4)	C7—N1—C6—C3	176.2 (3)
O2—S1—C2—C1	74.7 (3)	C4—C3—C6—O4	27.4 (5)
O3—S1—C2—C1	−157.0 (3)	S1—C3—C6—O4	−156.6 (3)
C3—S1—C2—C1	−42.6 (3)	C4—C3—C6—N1	−153.1 (4)
O2—S1—C3—C4	−108.1 (3)	S1—C3—C6—N1	22.9 (5)
O3—S1—C3—C4	123.0 (3)	C6—N1—C7—C12	138.0 (4)
C2—S1—C3—C4	7.8 (4)	C6—N1—C7—C8	−44.9 (6)
O2—S1—C3—C6	76.0 (3)	C12—C7—C8—C9	−1.3 (6)
O3—S1—C3—C6	−52.9 (3)	N1—C7—C8—C9	−178.4 (4)
C2—S1—C3—C6	−168.2 (3)	C7—C8—C9—C10	1.3 (7)
C1—O1—C4—C3	12.1 (6)	C8—C9—C10—C11	−0.6 (7)
C1—O1—C4—C5	−170.3 (3)	C9—C10—C11—C12	−0.3 (7)
C6—C3—C4—O1	−174.9 (3)	C8—C7—C12—C11	0.5 (6)
S1—C3—C4—O1	9.2 (5)	N1—C7—C12—C11	177.6 (4)
C6—C3—C4—C5	7.9 (6)	C10—C11—C12—C7	0.3 (6)
S1—C3—C4—C5	−168.0 (3)		

### Hydrogen-bond geometry (Å, °)

**Table:**

Cg is the centroid of the C7–C12 ring.

**Table:**

*D*—H···*A*	*D*—H	H···*A*	*D*···*A*	*D*—H···*A*
N1—H1A···O3	0.86	2.09	2.819 (4)	142
C2—H2B···O2^i^	0.97	2.50	3.274 (5)	137
C5—H5A···O4^ii^	0.96	2.52	3.422 (6)	157
C5—H5B···O4	0.96	2.37	2.786 (6)	106
C8—H8···O4	0.93	2.58	2.926 (5)	103
C9—H9···O4^iii^	0.93	2.49	3.419 (5)	175
C1—H1C···Cg^iv^	0.97	2.82	3.645	144

Symmetry codes: (i) −*x*+2, −*y*+2, −*z*; (ii) *x*+1, *y*, *z*; (iii) −*x*+1, −*y*+1, −*z*+1; (iv) *x*+1, *y*+1, *z*.
